# Sex- and age-related changes in GABA signaling components in the human cortex

**DOI:** 10.1186/s13293-018-0214-6

**Published:** 2019-01-14

**Authors:** Madhavi Pandya, Thulani H. Palpagama, Clinton Turner, Henry J. Waldvogel, Richard L. Faull, Andrea Kwakowsky

**Affiliations:** 1 Centre for Brain Research, Department of Anatomy and Medical Imaging, Faculty of Medical and Health Sciences, University of Auckland, Auckland, New Zealand; 2 Department of Anatomical Pathology, LabPlus, Auckland City Hospital, Auckland, New Zealand

**Keywords:** Sex difference, Aging, GAD, GABA_A_ receptor, GABA_B_ receptor, GABA transporter, Human brain

## Abstract

Gamma-aminobutyric acid (GABA) is the primary inhibitory neurotransmitter in the nervous system. Previous studies have shown fluctuations in expression levels of GABA signaling components—glutamic acid decarboxylase (GAD), GABA receptor (GABAR) subunit, and GABA transporter (GAT)—with increasing age and between sexes; however, this limited knowledge is highly based on animal models that produce inconsistent findings. This study is the first analysis of the age- and sex-specific changes of the GAD, GABA_A/B_R subunits, and GAT expression in the human primary sensory and motor cortices; superior (STG), middle (MTG), and inferior temporal gyrus (ITG); and cerebellum. Utilizing Western blotting, we found that the GABAergic system is relatively robust against sex and age-related differences in all brain regions examined. However, we observed several sex-dependent differences in GABA_A_R subunit expression in STG along with age-dependent GABA_A_R subunit and GAD level alteration. No significant age-related differences were found in α1, α2, α5, β3, and γ2 subunit expression in the STG. However, we found significantly higher GABA_A_R α3 subunit expression in the STG in young males compared to old males. We observed a significant sex-dependent difference in α1 subunit expression: males presenting significantly higher levels compared to women across all stages of life in STG. Older females showed significantly lower α2, α5, and β3 subunit expression compared to old males in the STG. These changes found in the STG might significantly influence GABAergic neurotransmission and lead to sex- and age-specific disease susceptibility and progression.

## Introduction

GABAergic interneurons account for approximately 20% of cortical neurons in the human brain that modulate neuronal activity via GABA based neuronal inhibition [1]. The balance between excitatory and inhibitory circuits is fundamental for all aspects of brain function. Existing data suggest that age and sex are significant contributors of altered neurotransmission between individuals and these differences might contribute to aging-related impairments and sex-specific vulnerability to disease conditions, for instance, depression, schizophrenia, presbycusis, and Alzheimer’s disease [2–12].

GABA is synthesized by glutamic acid decarboxylase (GAD) and is then recruited into synaptic vesicles. Following membrane depolarization, GABA is released into the synapse and binds to either ionotropic GABA_A_ receptors (GABA_A_Rs) or metabotropic GABA_B_ receptors (GABA_B_Rs). Released GABA is cleared from the synapse by membrane-bound GABA transporters, localized to neurons and astrocytes. Previous studies have reported aging-related alterations in the levels of both GAD isoforms, GAD65 and GAD67, in different species and brain areas. Using magnetic resonance spectroscopy several studies have found a reduction in concentration of GABA levels with age in animal models, nonhuman primates, and humans [13–15]. However, evidence suggesting loss of grey matter tissue fraction that causes an overall reduction in GABA concentrations confounds the correlation of age-related loss of GABA [16, 17]. In addition, alterations of GAD expression at the mRNA level and the protein level do not always follow the same trend and can be followed by changes in GABA level [18]. Furthermore, the literature also shows controversial results and species differences in GAD expression, some studies demonstrating an increase rather than a decrease in GAD levels in prefrontal cortical areas [2, 18, 19].

GABA_A_Rs are ligand-dependent Cl^−^ channel pores assembled from five subunits [20]. Over 20 GABA_A_R subunits have been identified; six alpha subunits (α1/2/3/4/5/6), three beta subunits (β1/2/3), three gamma subunits (γ1/2/3), delta (δ), theta (θ), epsilon (ε), pi (π), and rho (ρ1/2/3), forming many possible combinations of pentameric GABA_A_Rs [21–23]. Literature implies that the expression pattern of subunits is brain region specific and is involved in region-specific function [24, 25]. Therefore, previous studies hypothesized regional brain function loss of hearing impairment, learning, and memory deficit, as an implication of regional GABA_A_R subunit expression changes in aging [7, 8, 26, 27].

GABA_B_Rs are metabotropic, heterodimers formed by two subunits, GABA_B_R1 and GABA_B_R2, of which R1 binds GABA and R2 is associated with G proteins [28–30]. Evidence from animal studies demonstrates a loss of R1 subunit expression in the prefrontal cortex (PFC) and hippocampus in aged mice, leading to an overall downregulation of GABA_B_Rs, reduced inhibitory currents, and associated functional implications such as learning deficits and reduced memory formation [3, 31–33]. On the contrary, administration of a GABA_B_R antagonist demonstrated improvements in working memory in aged rats [19] and olfactory discrimination learning in mice [34]. Both GABA_B_R subunits show reduced expression in the rat PFC [19] and reduced GABA_B_R binding in the inferior colliculus and cortex with age [35–37]. These data indicate there is complex regulation of age-related GABA_B_R function.

The GABA transporters (GATs), GAT1/2/3 and betaine transporter 1 (BGT1), are present on interneurons and surrounding glial cells and regulate removal of GABA from the synaptic cleft [9, 38, 39]. Only few studies examined the age-related GAT changes; one study reported a decreased GAT1 expression in the rat medial PFC [19] and another in the human frontal cortex [40]. Age-related reduction in expression of GAT1 and GAT2 were also observed in the rhesus macaque visual cortex [41] corresponding with age-related visual retrogression in these primates [42].

The confounding issue of sex-based variability in the brain at the molecular and cellular level is well established. Gene and hormonal differences are the leading cause of behavioral and physiological changes observed between sexes with few studies suggesting a magnitude of asymmetrical sex-led differences in the GABAergic system [41, 43]. The changes in hormonal levels (estradiol and progesterone) during the menstrual cycle have been suggested as causative of fluctuation of brain GABA levels in healthy females [44, 45]. Furthermore, the GABA level fluctuations coincide with behavioral changes such as mood, cognitive function, and physical symptoms [44–46]. Gonadal steroidal hormones, such as estrogens, are known manipulators of synaptic transmission through genomic mechanisms as well as rapid alteration in cell to cell communication [47–53]. Estrogens also regulate the release of GABA and induce bursts in GABA_A_R-dependent inhibitory postsynaptic currents in gonadotropin-releasing hormone neurons [49, 54, 55]. Another ovarian hormone progesterone and its metabolite allopregnanolone are also regulators of inhibitory neurotransmission through their influence on GABA_A_R [5, 56, 57]. Studies show that short-term exposure to allopregnanolone leads to upregulation of the α4 subunit [58]. The ability of ovarian hormones to regulate GABA_A_R subunit composition and alter their function, pharmacology, and GABA-gated current is another mechanism that could lead to changed neurotransmission. Predominantly, these hormonal changes are drastic in females during puberty, through the menstrual cycle, pregnancy, and post-menopausal period, and therefore, hypothetically, females are more susceptible towards hormonal driven changes in the GABAergic system. These findings suggest a differential mechanism and response in males and females towards changing hormonal levels throughout stages of life and aging.

Sex and age bias has been observed in many neurological disorders such as Alzheimer’s disease and depression disorder [5, 59–63] and has also been linked to the GABAergic system [6, 27, 60, 64, 65]. Therefore, a thorough investigation is required to identify the link between sex and age and the GABAergic changes observed in these and other neurological conditions.

This study is the first comprehensive analysis of the sex- and age-specific expression of GABA signaling components in the human neocortical areas; primary, secondary, and association areas from each lobe; and cerebellar cortex. In the present study, we observed only a few alterations in the expression of GAD, GABA_A_R, GABA_B_R subunits, and transporters GAT-1/3 in the primary sensory and motor cortices, middle (MTG) and inferior temporal gyrus (ITG), and cerebellum, except the superior temporal gyrus (STG) that displayed numerous sex- and age-related expression changes, mainly affecting GAD65 and the GABA_A_Rs.

## Methods

### Human brain tissue preparation and neuropathological analysis

This study was conducted at the University of Auckland, Centre for Brain Research. The tissue was acquired through a donor program to the Neurological Foundation of New Zealand Human Brain Bank, and the procedures were approved by the University of Auckland Human Participant’s Ethics Committee (Approval number: 011654). Seven control younger females (YF, 51.7 years ± 5.1 years), six younger males (YM, 47.5 years ± 1.5 years), eight older females (OF, 76 years ± 1.3 years), and seven older males cases (OM, 80 years ± 1.5 years) with a maximum post-mortem time of 26 h (Table 1) were chosen. Processing of tissue followed the procedure described previously [66]. Firstly, the brain was dissected in half separating the hemispheres; the left hemisphere of the brain was cut into anatomical blocks, freshly frozen, and stored at − 80 °C. All cases included in this study had no history of any primary neurodegenerative, psychiatric disorder, neurological disease abnormalities, or excessive alcohol consumption. Standard pathological sections from all cases, including the middle frontal, middle temporal, and cingulate gyrus; hippocampus; caudate nucleus; substantia nigra; locus coeruleus; and cerebellum, were examined and confirmed as pathologically normal by a neuropathologist.

**Table 1:** Human brain case details for all experimental groups. YF, younger female, OF, older female, YM, younger male, OM, older male, PM, post-mortem

Case	Age	Sex	PM delay	Cause of death	Weight (g)	Classification
110	83	F	14	Aortic aneurysm	1200	OF
111	46	M	10	Coronary artery disease	1424	YM
112	79	M	8	Bleeding stomach ulcer	1190	OM
121	64	F	6.5	Pulmonary embolism	1205	YF
122	72	F	9	Emphysema	1230	OF
123	78	M	7.5	Abdominal aortic aneurysm	1260	OM
124	49	M	13	Ischemic heart disease	1495	YM
126	36	F	11	Asphyxia	1320	YF
127	59	F	21	Pulmonary embolism	1310	YF
128	34	F	18.5	Myocardial infarction	1140	YF
129	48	M	12	Pulmonary embolism	1318	YM
131	73	F	13	Ischemic heart disease	1210	OF
132	63	F	12	Rupture aorta	1280	YF
137	77	F	21	Coronary atherosclerosis	1227	OF
152	79	M	18	Congestive heart failure	1425	OM
156	89	M	19	Atherosclerosis	1430	OM
159	53	M	16.5	Ischemic heart disease	1215	YM
165	43	F	26	Nitrogen poisoning	1318	YF
169	81	M	24	Asphyxia	1225	OM
181	78	F	20	Aortic aneurysm	1292	OF
189	41	M	16	Asphyxia	1412	YM
190	72	F	19	Myocardial infarction	1264	OF
202	83	M	14	Abdominal aortic aneurysm	1245	OM
209	48	M	23	Ischemic heart disease	1470	YM
238	63	F	16	Aortic aneurysm	1324	YF
241	76	F	12	Metastatic cancer	1094	OF
243	77	F	13	Ischemic heart disease—coronary atherosclerosis	1184	OF
244	76	M	16	Ischemic heart disease—coronary atherosclerosis	1508	OM

### Western blotting

The fresh human cortical tissue samples were collected from the regions of interest (sensory and motor cortex; cerebellum; superior, middle, and inferior temporal gyrus) using a cryostat (CM3050, Leica Microsystems, Germany) at 60-μm thickness on glass slides. The grey matter tissue was collected with a blade, homogenized in a buffer containing 0.5 M Tris, 100 mM EDTA, 4% SDS, pH 6.8, and protein extracts prepared using 0.5-mm glass beads (Mo BIO, USA) and a Mini Bullet Blender Tissue Homogenizer (Next Advance, Inc., New York, USA) at speed 8 for 8 min. The homogenates were incubated for 1 h on ice, then centrifuged at 10,000 rpm for 10 min; the supernatant collected and stored at − 20 °C. The protein concentration of the samples was measured using detergent-compatible protein assay (DC Protein assay, 500-0116, Bio-Rad, Hercules, CA, USA), following the manufacturer’s instructions. Protein samples from each case were randomized, by a person not involved in the study, and numbered from 1 to 24. Twenty micrograms of each protein extract was run on a gradient SDS PAGE gel (NU PAGE 4–12% BT 1.5, NP0336BOX, Life technologies, California, USA) and then blotted. Proteins were separated in XCell SureLock Mini-Cell system (Invitrogen, Victoria, Australia) and transferred onto nitrocellulose membranes using XCell Blot Module (Invitrogen, Victoria, Australia). Three molecular weight ladders, Molecular weight, SeeBlue or Magic mark (Life technologies, California, USA), were also loaded in gels as verification of labeled band size. Membranes were blocked with Odyssey blocking buffer (LI-COR Biosciences, USA) at room temperature for 30 min, followed by incubation with the primary antibodies (Table 2), at 4 °C overnight. The following day membranes were washed 3 × 10 min in Tris-buffered saline pH 7.6, 0.1% Tween (TBST) and incubated with an appropriate IRDye (1:10,000, goat anti-rabbit IRDye®680RD, 926-68071, RRID:AB_10956166; goat anti-mouse IRDye®800CW, 926-32210, RRID:AB_621842; donkey anti-goat IRDye®800CW, 926-32214, RRID:AB_621846; LI-COR Biosciences, Germany) secondary antibody for 1 h at room temperature. Membranes were washed and scanned on an Odyssey Infrared Imaging System (LI-COR Biosciences, USA).

**Table 2:** Primary antibodies used in this study

Antigen	Host species, source, catalogue, number	Concentration	Immunogen
Anti-GABA_A_R α1	Rabbit, Alomone, AGA-001	1:1000	Peptide QPSQDELKDNTTVFTR
Anti-GABA_A_R α2	Rabbit, Alomone, AGA-002	1:200	Peptide (C)TPEPNKKPENKPA
Anti-GABA_A_R α3	Rabbit, Alomone, AGA-003	1:200	Peptide QGESRRQEPGDFVKQ
Anti-GABA_A_R α5	Rabbit, Thermo Fischer, PA5-31163	1:200	Recombinant fragment corresponding to amino acids 142 and 379 of human GABA_A_R α5
Anti-GABA_A_R γ2	Goat, Santa Cruz, SC-131935	1:100	Extracellular domain of human GABA_A_R γ2
Anti-GABA_A_R β3	Mouse, Novus, NB-1-47,613	1:500	Peptide corresponding to amino acids 370-433 of mouse GABA_A_R β3
Anti-GAD65	Mouse, Millipore, MAB351	1:1000	Purified rat brain glutamic acid decarboxylase
Anti-GAD67	Mouse, Millipore, MAB5406	1:200	Recombinant GAD67 protein
Anti-GABA_B_R R2	Mouse, NeuroMab, 75-124	1:400	Fusion protein amino acids 861-912 of rat GABA_B_R R2
Anti-GAT1	Rabbit, Alomone, AGT-001	1:100	Peptide (C)ERNMHQMTDGLDK
Anti-GAT3	Rabbit, Alomone, AGT-003	1:100	Peptide (C)REARDKAVHERGH
Anti-β-actin	Rabbit, Abcam, ab8227	1:1000	Human β-actin amino acids 1-100
Anti-β-actin	Mouse, Abcam, ab6276	1:1000	Peptide DDDIAALVIDNGSGK

### Nissl staining

Nissl staining was performed for identification of the sensory and motor cortex regions on each block. Fresh frozen section were stained with a cresyl violet solution (2% Cresyl violet in 0.1 M glacial acetic acid and 0.0136 M sodium acetate solution) for a period of 45 min, mounted onto glass slides, dried, dehydrated through a graded series of ethanol, and cleared in xylene. Sections were examined using a Leica (Wetzlar, Germany) DMRB light microscope. Tissue sections were examined for features such as cortical thickness and the presence of large motor neurons.

### Imaging and analysis

Odyssey Infrared Imaging System (LI-COR Biosciences, USA)-based detection of immunofluorescence signal was carried out at 680-nm and 800-nm spectrum. The analyses were conducted using the Image Studio Lite software (version 5.2, LI-COR Biosciences, USA) to measure signal intensities of each sample and were normalized to β-actin. To examine the averaged signal intensity differences between groups (younger females (*n* = 6) vs younger males (*n* = 6); younger females (*n* = 6) vs older females (*n* = 6); younger males (*n* = 6) vs older males (*n* = 6); older females (*n* = 6) vs older males (*n* = 6)), a non-parametric Kruskal-Wallis test was used. Data in all experiments was expressed as mean ± SEM. All statistical analyses were conducted using Prism (version 6; GraphPad Software) with a value of *p* < 0.05 considered significant.

## Results

**Fig. 1:**
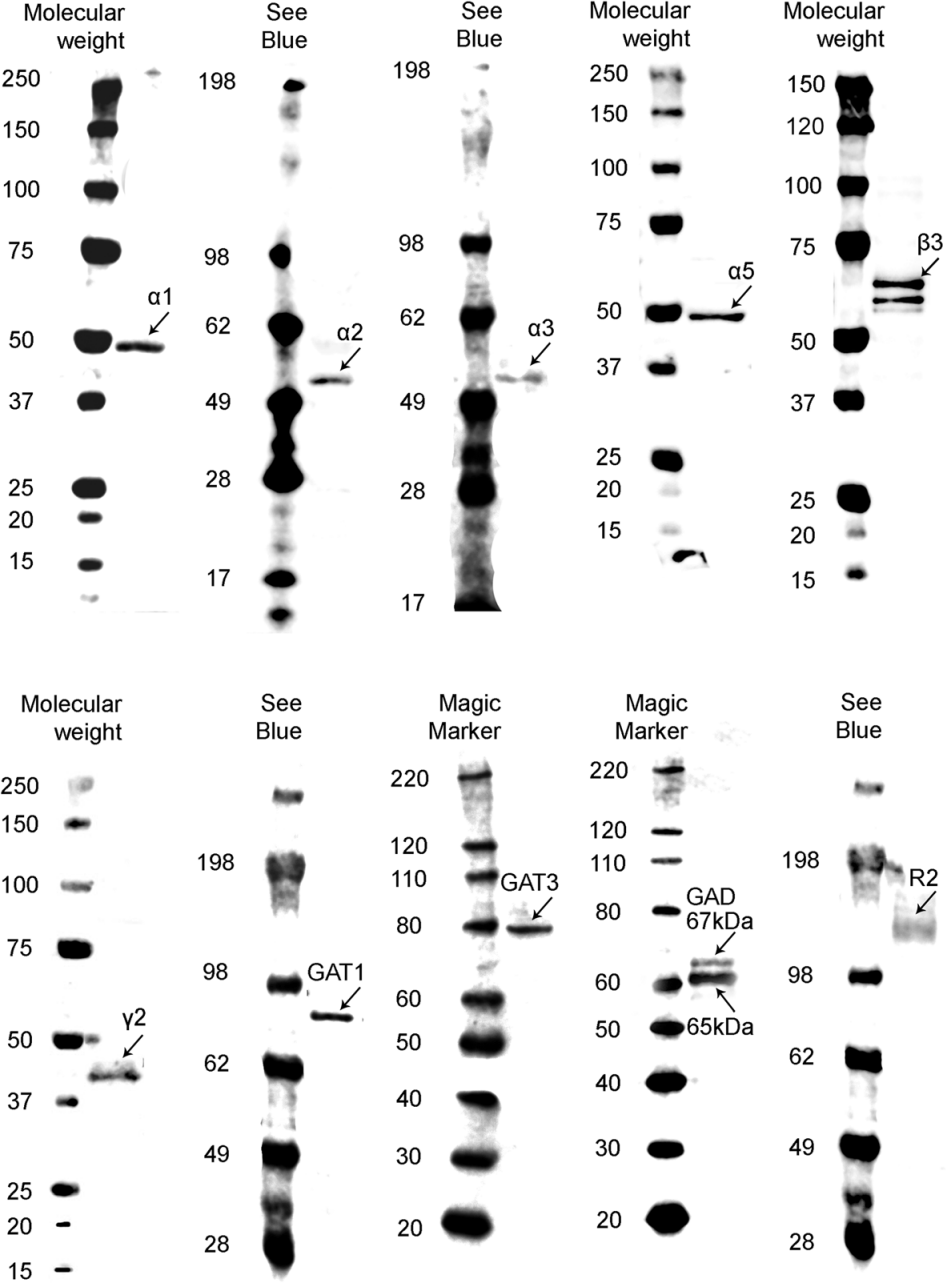
Western blot against human brain protein homogenates probed with GABA_A_ receptor α1, α2, α3, α5, β3, and γ2 subunit, GAT1, GAT3, GAD65 and GAD67 and GABA_B_R R2 subunit antibodies. Each lane has 20 μg of protein loaded. Observed band sizes: α1, α2: ~ 52 kDa; α3, α5: ~ 55 kDa; β3: ~ 63 kDa; γ2: ~ 44 kDa; GAT1: ~ 85 kDa, GAT3: ~ 80 kDa; GABA_B_R R2: ~ 120 kDa

**Fig. 2:**
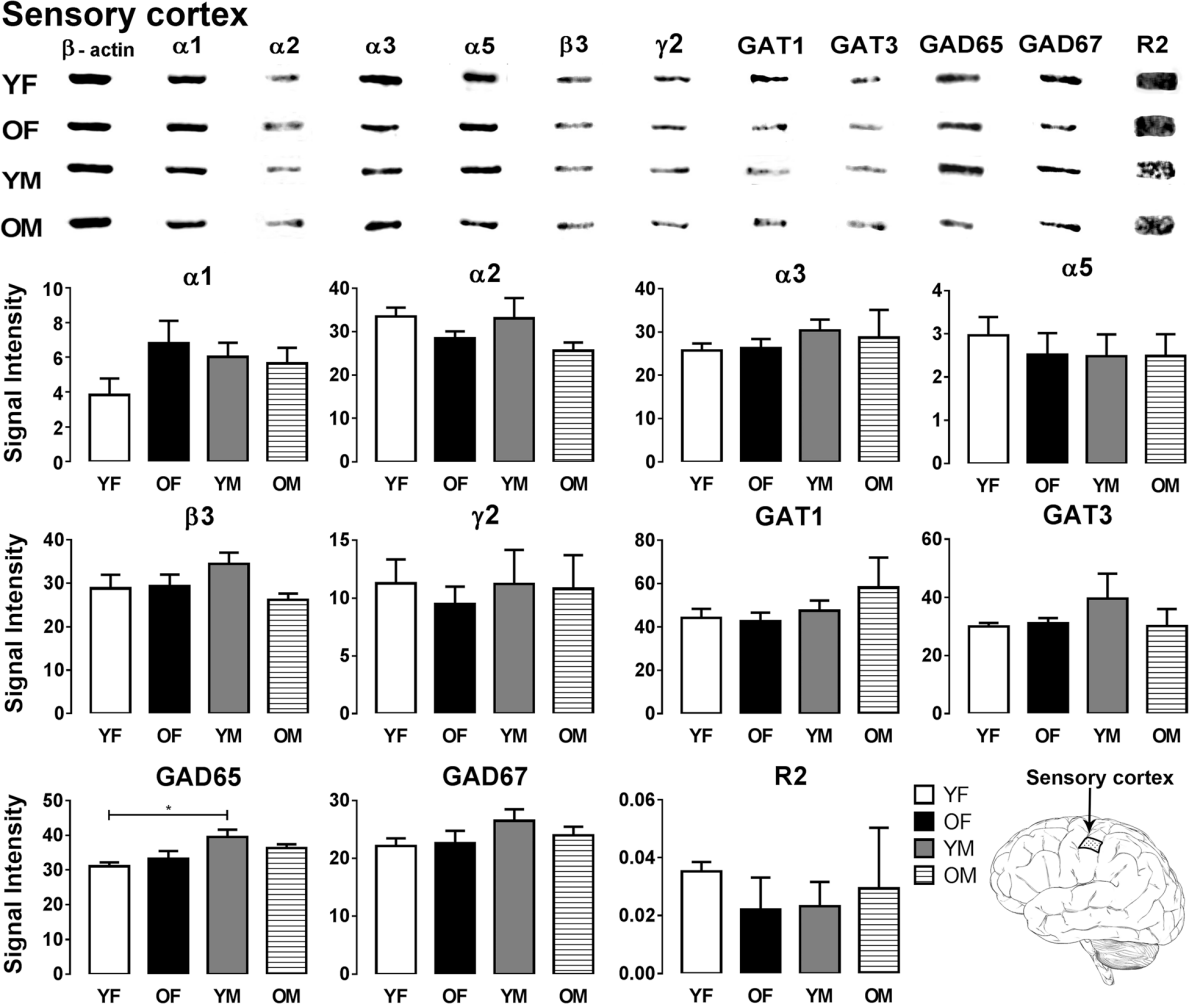
Representative immunoreactive Western blot bands from younger female (YF), older female (OF), younger male (YM), and older male (OM) sensory cortex homogenates following incubation with antibodies to the GABA_A_R subunits α1, α2, α3, α5, β3, and γ2 and GABA_B_R subunit R2, GAT1, GAT3, GAD65, and GAD67 (**a**) and corresponding signal intensity graphs (**b**). Signal intensity for each GABA signaling component Western blot band was measured and normalized to their corresponding β-actin signal for each age group. The data is graphed as mean ± SEM (*n* = 6; Kruskal-Wallis test; p* < 0.05)

**Fig. 3:**
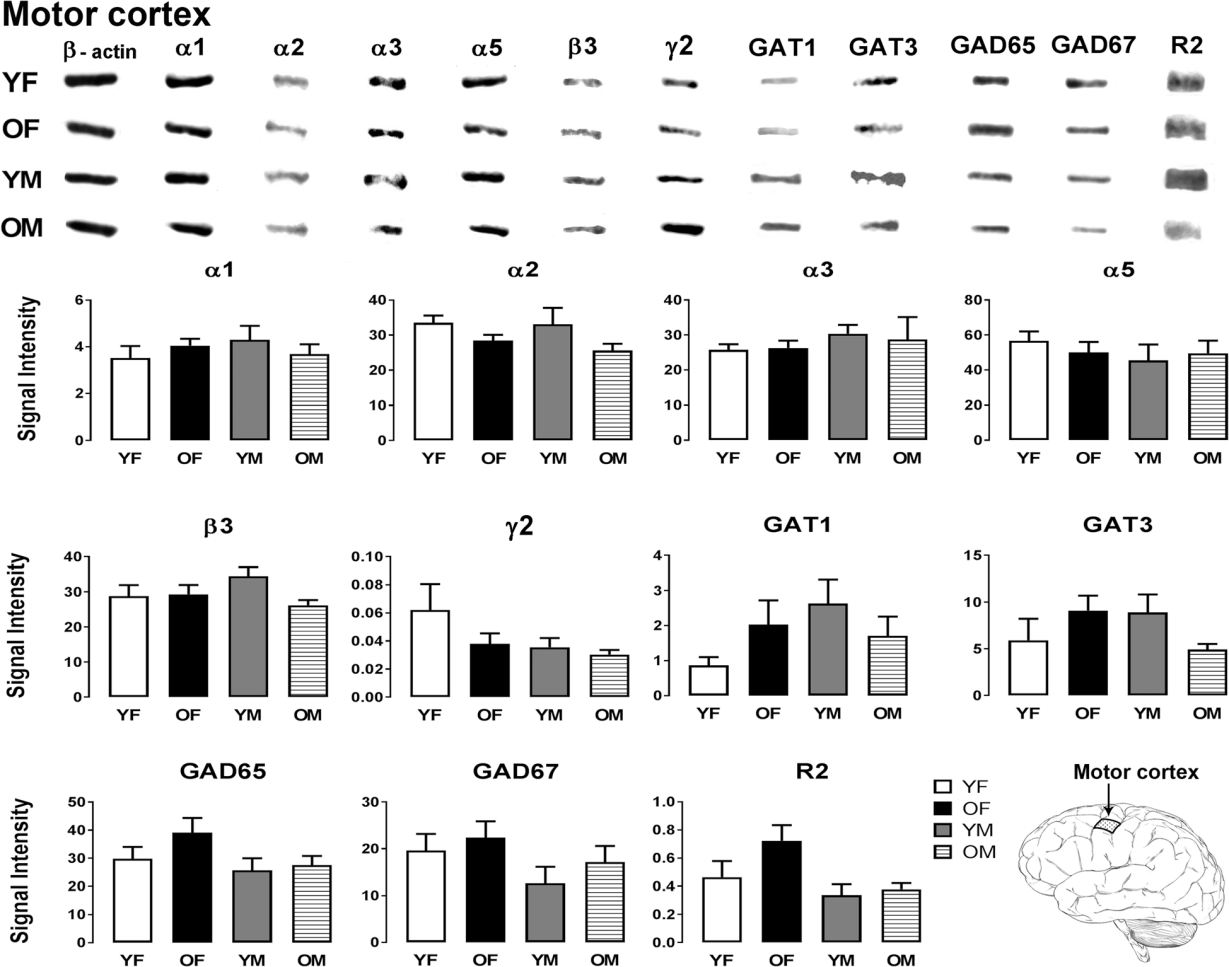
Representative immunoreactive Western blot bands from younger female (YF), older female (OF), younger male (YM), and older male (OM) motor cortex homogenates following incubation with antibodies to the GABA_A_R subunits α1, α2, α3, α5, β3, and γ2 and GABA_B_R subunit R2, GAT1, GAT3, GAD65, and GAD67 (**a**) and corresponding signal intensity graphs (**b**). Also, see figure legend on Fig. 2 for details

**Fig. 4:**
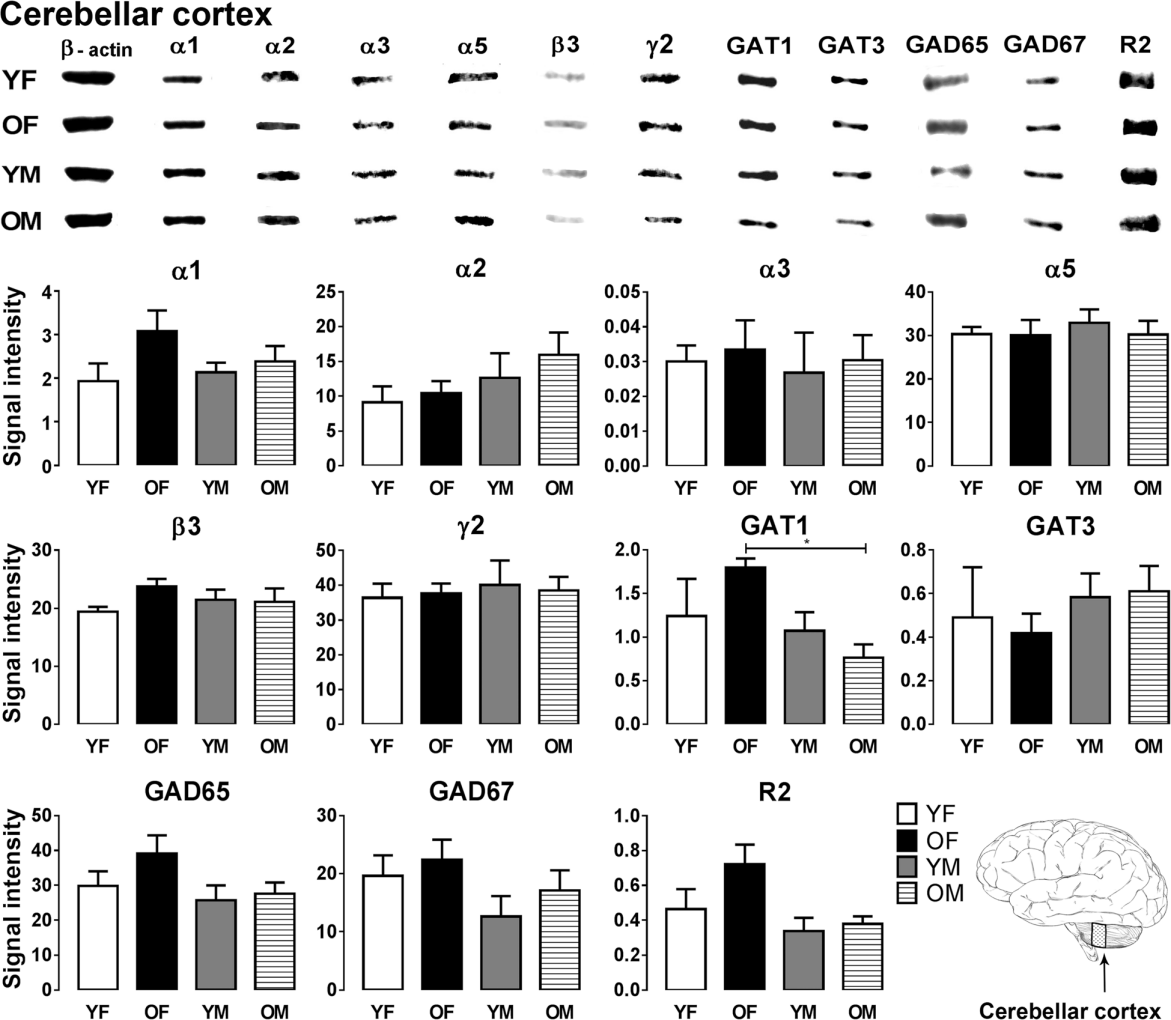
Representative immunoreactive Western blot bands from younger female (YF), older female (OF), younger male (YM), and older male (OM) cerebellum homogenates following incubation with antibodies to the GABA_A_R subunits α1, α2, α3, α5, β3, and γ2 and GABA_B_R subunit R2, GAT1, GAT3, GAD65, and GAD67 (**a**) and corresponding signal intensity graphs (**b**). Also, see figure legend on Fig. 2 for details

**Fig. 5:**
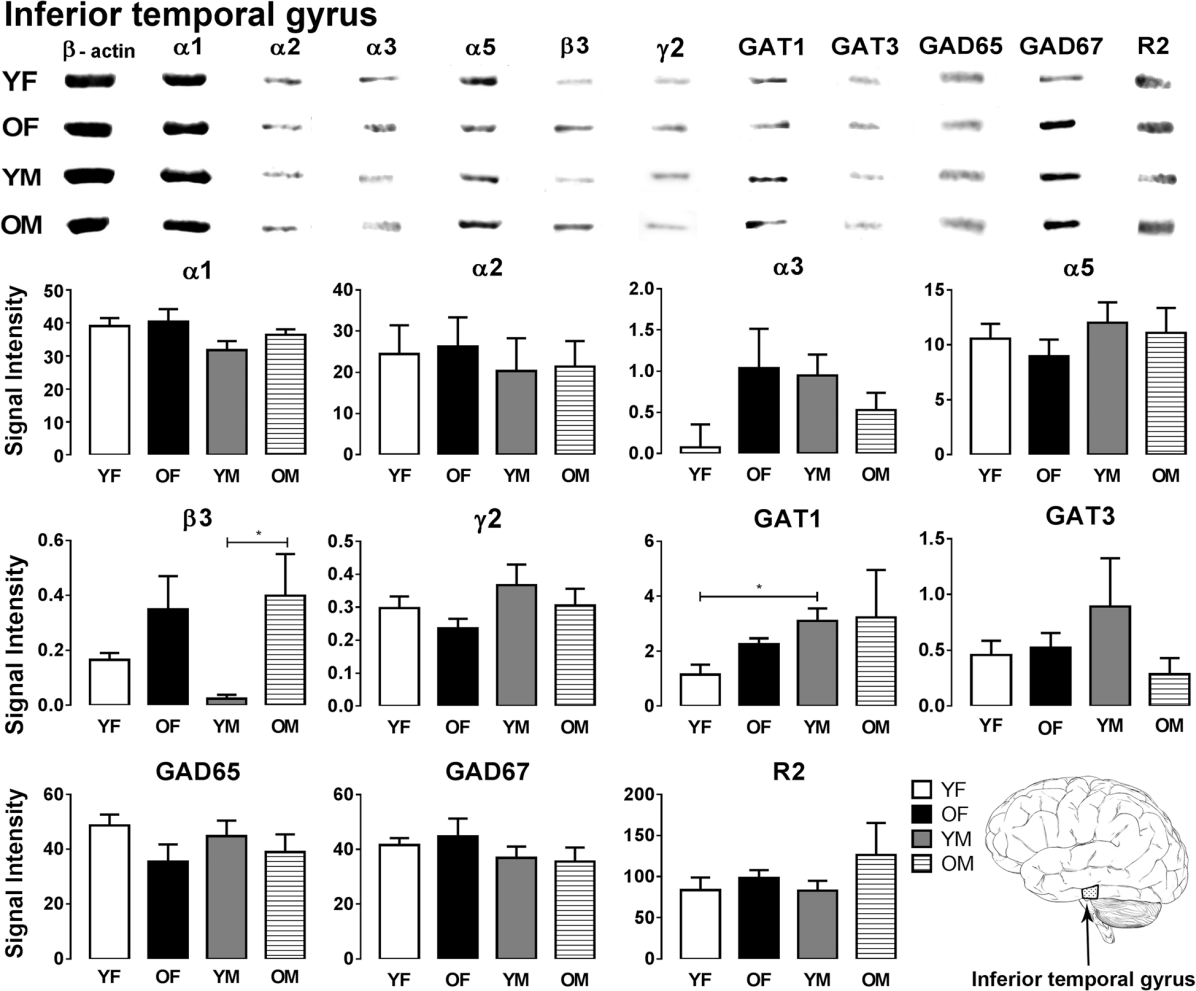
Representative immunoreactive Western blot bands from younger female (YF), older female (OF), younger male (YM), and older male (OM) inferior temporal gyrus homogenates following incubation with antibodies to the GABA_A_R subunits α1, α2, α3, α5, β3, and γ2 and GABA_B_R subunit R2, GAT1, GAT3, GAD65, and GAD67 (**a**) and corresponding signal intensity graphs (**b**). Also, see figure legend on Fig. 2 for details

**Fig. 6:**
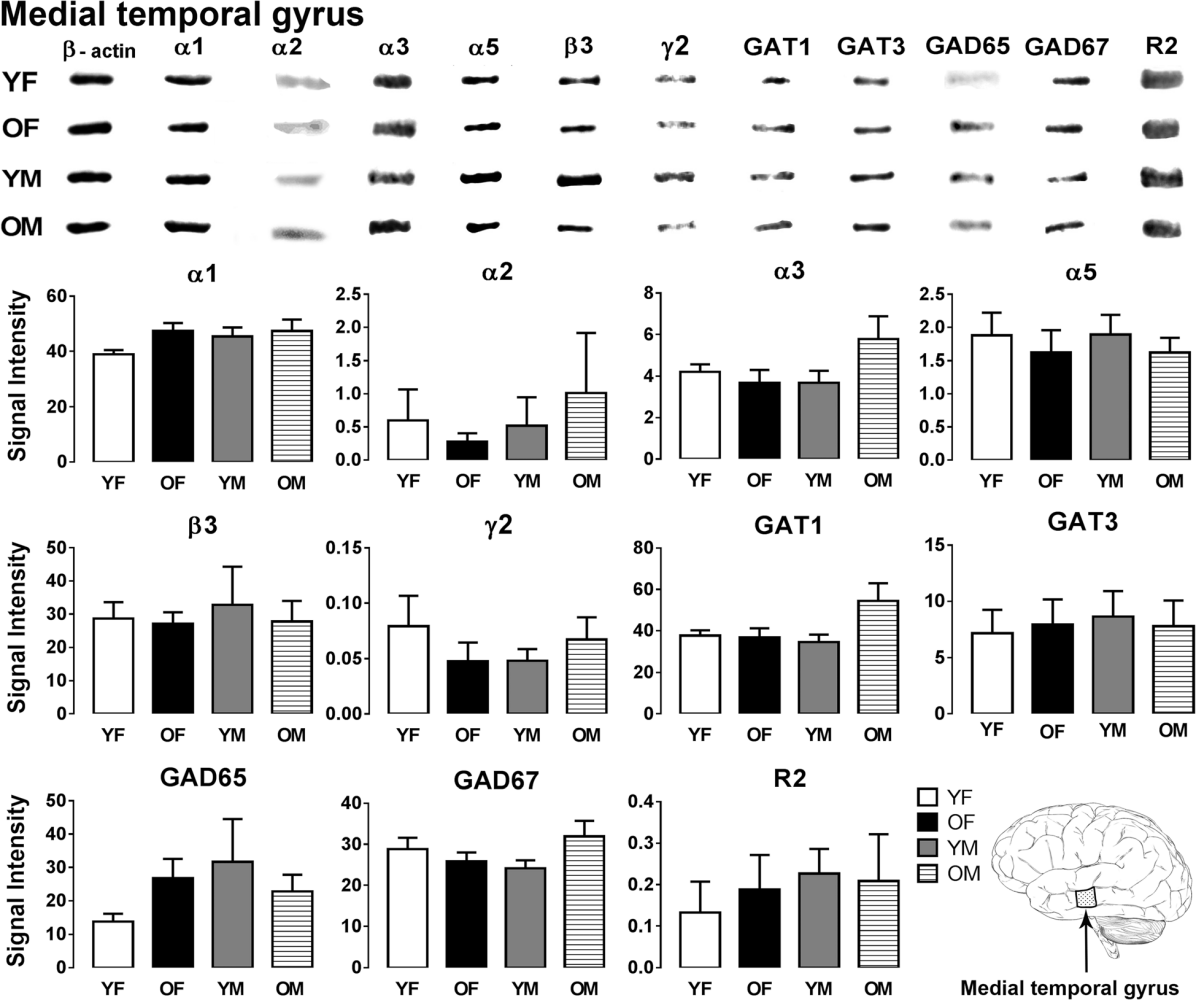
Representative immunoreactive Western blot bands from younger female (YF), older female (OF), younger male (YM), and older male (OM) medial temporal gyrus homogenates following incubation with antibodies to the GABA_A_R subunits α1, α2, α3, α5, β3, and γ2 and GABA_B_R subunit R2, GAT1, GAT3, GAD65, and GAD67 (**a**) and corresponding signal intensity graphs (**b**). Also, see figure legend on Fig. 2 for details

**Fig. 7:**
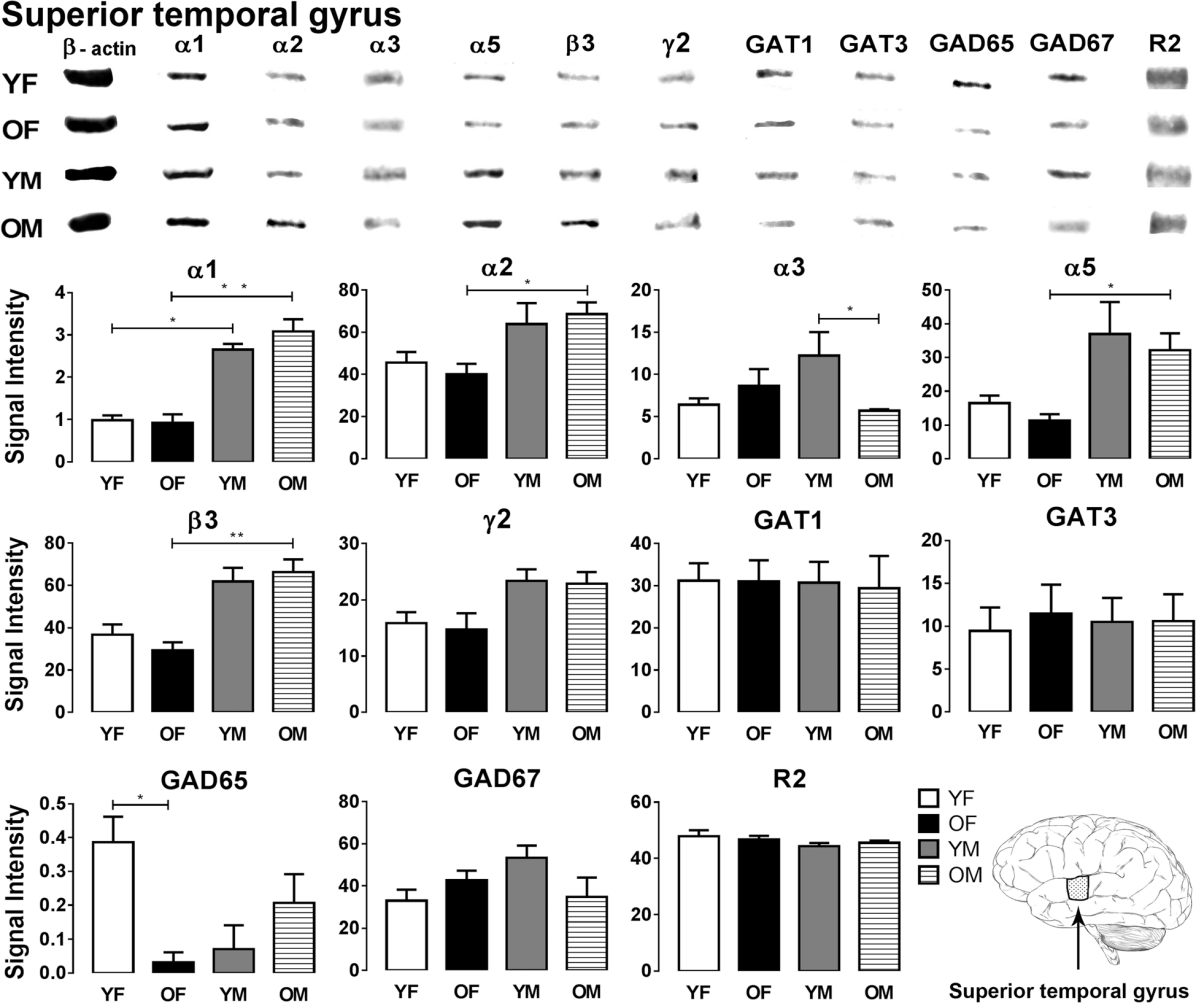
Representative immunoreactive Western blot bands from younger female (YF), older female (OF), younger male (YM), and older male (OM) superior temporal gyrus homogenates following incubation with antibodies to the GABA_A_R subunits α1, α2, α3, α5, β3, and γ2 and GABA_B_R subunit R2, GAT1, GAT3, GAD65, and GAD67 (**a**) and corresponding signal intensity graphs (**b**). Also, see figure legend on Fig. 2 for details

The expression levels of GABA signaling components, GABA_A_R subunits (α1/2/3/5, β3 and γ2), GABA_B_R subunit R2, GAT1, GAT3, GAD65, and GAD67, were examined by Western blotting in the sensory and motor cortices, cerebellum, and human inferior (ITG), middle (MTG), and superior (STG) temporal gyrus (Figs. 1, 2, 3, 4, 5, 6, and 7).

In the sensory cortex, most GABA signaling components showed similar expression across the four age and gender groups; however, a significant sex-related GAD65 expression difference was observed (Fig. 2). GAD65 expression in young males was significantly higher compared to young females (*p* = 0.0189). The motor cortex did not show significant changes in the expression level of any of the GABA signaling components examined between the four groups (Fig. 3).

In the cerebellum, most GABA signaling components were well preserved during aging. One significant sex-related GAT1 expression difference observed was that older females showed significantly higher expression of GAT1 compared to the older male group (*p* = 0.0249) (Fig. 4).

In the ITG, most GABA signaling components displayed similar expression level across all the groups (Fig. 5). However, significant age-related alteration was observed in GABA_A_R β3 subunit expression as older males show significantly higher β3 subunit levels compared to young males (*p* = 0.035). Also, GAT1 expression was significantly higher in younger males compared to younger females (*p* = 0.024) (Fig. 5).

In the MTG, all GABA signaling components were well preserved across the examined four groups as no significant changes were observed (Fig. 6).

In the STG, significant sex-related changes were observed in expression of the GABA_A_R α1 subunit, as males show much higher expression of this subunit compared to females in both age groups (*p* = 0.049 YF vs YM; *p* = 0.018 OF vs OM) (Fig. 7). Similarly, expression of GABA_A_R α2, α5 and β3 subunits is significantly higher in older males compared to the older female group (*p* = 0.040, α2; *p* = 0.010, α5; *p* = 0.004, β3) (Fig. 7). The GABA_A_R α3 subunit and GAD65 showed age-specific expression changes in the STG. The α3 subunit shows significantly lower expression in older males compared to younger males (*p* = 0.035) (Fig. 7). GAD65 expression was significantly higher in younger females compared to older females (*p* = 0.019) (Fig. 7). In the STG, all other GABA signaling components displayed similar expression level across all the groups (Fig. 7).

## Discussion

In this study, we report that the GABAergic system in the human primary sensory and motor cortices, cerebellum, and ITG and MTG is generally well protected against sex- and age-related alterations. GABA_B_Rs are especially robust, with no expression level differences found between groups in any of the brain regions examined. The major finding of our study is the presence of strong sex-related differences in the STG, as well as a few minor differences in the other cortical areas examined, supporting the importance of accounting for sex differences between groups in future studies and the development or prescription of treatment therapies. The STG also displayed age-specific GABA_A_R subunit expression decrease in older males and GAD65 level decrease in older females. Previous literature suggests that GABAR and GAD level alterations may subsequently lead to compensatory changes in order to maintain homeostasis, and this may affect regional network functionality [2–9, 67–69]. This is the first study to explore the sex- and age-specific expression of GABA signaling components in the aforementioned brain regions and to predict the potentially resulting functional alterations.

Importantly, of all the cortical regions examined, the temporal lobe is the only one that displays an age-related decrease in GABA signaling component expression besides the observed sex-specific GABA_A_R subunit alterations within the STG. The temporal lobes have a unique architecture and functional characteristics that make them particularly vulnerable to certain disease processes. They are interconnected through the anterior commissure, the corpus callosum, and the hippocampal commissure, and these connections are among the underlying mechanisms that contribute to disease processes. The optic tract and radiation may also spread pathology from the optic chiasm to both temporal lobes via Meyer’s loop that passes through the STG [70, 71]. The STG is also the most proximal gyrus of the temporal lobe to the anterior commissure and has direct connections with the corpus callosum [71, 72]. Some disease processes have selective affinity to specific areas of the temporal lobe due to selective limbic system vulnerabilities that might be immune-mediated, related to sensitivity to hypoxia and aging [70, 73, 74]. The temporal lobe is the location of the primary auditory cortex, which is important for the processing and interpretation of sounds and language. This lobe integrates auditory, sensory, visual, and limbic function, including memory processing and formation.

Our current knowledge of GABAergic sex- and age-related alterations across different regions of the human brain, including the temporal lobe, is limited. Previous studies have reported conflicting findings and species differences in GAD and GABA level changes in prefrontal cortical areas [2, 18, 19]. However, developmental GABAergic changes in the human visual cortex and across the lifespan are relatively well documented [75]. Our study showed significant reductions in GAD65 expression in the STG with age in females. This is in agreement with the previously reported slight decline in GAD65 expression in the visual cortex in older adults (> 55 years) [75] and rhesus macaque (19–20 years) [41]. In the STG, we observed a small non-significant increase in GAD67 expression in the older female group compared with the younger group. A similar trend towards an increase in GAD67 levels has been reported in the humans [75] and a significant increase in GAD67 has also been observed in the rhesus macaque visual cortex [41]. While previous GAD65 knock-out mouse studies have demonstrated no GAD67 or GABA level deficits [76, 77], several studies suggest that GAD65 loss may result in region-specific hyperexcitability and functional implications, such as susceptible to seizures [76–80]. GAD65 is involved in rapid GABA release and provides most of the GABA for neurotransmitter release, and under pathological conditions, GAD67 could play a similar role. Previous studies imply a consequential increase in vesicular GABA transporter (VGAT) expression in response to GAD65 knock-out [81]. Upregulation of VGAT, however, only partially contributes to increased GABA uptake into vesicles and GAD67 might play an important compensatory role [81]. The age-related decrease in the expression of GAD65 in the STG and visual cortex and the reciprocal increase in GAD67 expression might be the result of a shift in the expression of GAD67 to compensate for the effect of GAD65 loss on GABA synthesis [82, 83]. However, the functional implications of this age-related GAD65 expression decrease in the older female group are difficult to predict, and further functional studies will be required to understand the physiological consequences of this change. However these changes might underlie age-dependent disease susceptibility and influence the progression of Alzheimer’s disease, epilepsy, or schizophrenia, conditions in which the fine balance of excitation and inhibition is impaired.

GABA_A_R subunit densities exhibit varying expression profiles in different regions of the human cerebral cortex [24]. As mentioned earlier, our study is the first to examine age- and sex-related changes in the expression of GABA_A_R subunits in the temporal gyri, and we demonstrate that the STG displays the greatest magnitude of age- and sex-related changes in GABA_A_R subunit expression. Importantly, only a few previous studies have reported sex-specific changes in the expression of GABA_A_R subunits in the primate brain [41], and the lack of human data warrants further research in this area. Examination of the STG demonstrated a sex-related difference in β3 subunit expression; older males showed significantly higher expression compared with older females, and a similar trend has been observed between younger females and younger males as well. In the ITG, the β3 subunit displayed age-specific expression changes; older males had significantly higher β3 subunit levels compared with younger males, with a similar trend between the younger and older female groups. Previous results from a human dorsolateral PFC study showed a relatively stable β3 subunit expression with aging, but the average age of the oldest age group in the study was only 43 years of age [84]. However, we can also deduce that the differential expression pattern observed in our study might be due to regional differences in the regulation of subunit expression. The α3 subunit also shows age-related expression changes in the STG; in younger males, the expression was significantly higher compared with older males. We observed a sex difference in α1 subunit expression in the STG as males show significantly higher expression than females in both age groups. Furthermore, α2, α5, and β3 subunit expression is significantly higher in older males compared with older females, and a similar trend has been also observed between the younger male and female groups. The STG contains the transverse gyrus of Heschl and Wernicke’s area that are involved in the processing of auditory sensory information. Based on all the expression changes found in the STG, we might suspect a sex- and age-related influence on auditory function. Evidence based on animal experiments shows that synaptic inhibitory mechanisms in the auditory cortex are particularly vulnerable to aging [26]. Results from in situ hybridization studies show an age-related reduction in the GABA_A_R α1 subunit transcript across all layers of the auditory cortex. An age-related increase in α3 subunit expression was observed in a subset of layers of the auditory cortex (layers II and III). The GABA_A_R β1, β2, γ1, γ2s, and γ2L subunits also showed age-related declines at the mRNA and protein levels [26]. Other changes, such as the loss of GAD65 and GAD67 in the auditory cortex and increased spontaneous neuronal activity in the inferior colliculi and auditory cortex, have also been observed, leading to a synergistic loss of GABA signaling components and impaired auditory function in aged rats [85, 86]. As mentioned before, we also observed GAD65 loss in the STG, but GAD67 levels are well preserved in the human secondary auditory cortex. The discrepancy between the rat and human data might be the result of species differences or the techniques used, as mRNA expression is not necessarily proportional to protein levels.

Data from sex-differentiated studies suggest that the etiology and progression of age-related hearing loss (presbycusis) differs in males and females with age [12]. The magnitude of sex-related GABA_A_R subunit changes observed in the STG in this study might contribute to sex-specific hearing loss with aging, besides many other possible factors. The differential expression patterns of GABA_A_R subunits affects GABA binding affinity and the downstream function of the receptor, altering neuronal excitability and the activity of neuronal networks [22]. We hypothesize that the GABA signaling component expression changes in the STG might contribute to alterations in higher auditory information processing and have an effect on memory formation and processing. However, peripheral hearing impairment might also lead to central molecular and cellular changes, including the GABAergic system. As age-related hearing loss displays sex-specific differences, these alterations might explain why the STG is the area most vulnerable to sex-specific GABAergic changes.

The STG is strongly implicated in the pathophysiology of schizophrenia, particularly with regard to auditory hallucinations [87–90]. A significant (30%) increase in the binding of [3H] muscimol in the STG has been observed in schizophrenia patients compared with control subjects, suggesting an increase in GABA_A_R density in the STG in this disease [91]. Previous studies demonstrated that working memory dysfunction in schizophrenia is mediated by altered GABAergic neurotransmission in certain dorsolateral prefrontal cortex microcircuits. Subjects with schizophrenia exhibited expression deficits in GABA signaling-related mRNA transcripts; the downregulation of GAT1 in the presynaptic terminals of parvalbumin-containing chandelier neurons [92]; the upregulation of the GABA_A_R α2 subunit in the postsynaptic axon initial segments of pyramidal neurons [93]; deficits in GAD67 and VGAT [94]; neuropeptides (somatostatin, neuropeptide Y and cholecystokinin); and the GABA_A_R α1, α4, β3, γ2, δ [94, 95] and α5 subunits [96]. Age- and sex-related differences are present in schizophrenia, but the mechanisms underlying these require further investigation [11, 97–102]. The sex- and age-specific differences in GABA_A_R and GAD65 levels observed in the STG in this study might play a crucial role in the pathogenesis of schizophrenia and in disease susceptibility, but a direct link will have to be established.

GABA_A_R subunit expression changes have been reported in other disease conditions such as epilepsy and Alzheimer’s disease [27, 103]. Several studies have reported sex-specific susceptibility to the development of specific epilepsy subtypes, particularly in temporal lobe epilepsies in females [104–106]. The impairment of GABA_A_ receptor-mediated inhibition causes an increase in neuronal excitability and plays a critical role during epileptogenesis [107, 108]. The sex-specific reduction in GABA_A_R α1, α2, α5, and β3 subunit expression observed in females in this study might be a factor underlying their higher susceptibility for temporal lobe epilepsies. Some sex hormones and neuroactive steroids are potent activators of GABA_A_Rs and can therefore change the expression of some GABA_A_R subunits [57, 58, 108, 109]. Interestingly, sex steroids do not seem to influence the expression of the examined GABA_A_R subunits during aging in females; despite the fact that some of the younger females might have been pre-menopausal while others post-menopausal, the hormonal levels did not lead to greater variation in GABA_A_R subunit expression levels and we have not observed any subunit alterations between the younger and older female group*.* Animal studies have demonstrated GABA_A_R subunit expression alterations during the estrus cycle and pregnancy, although these studies have mostly implicated extrasynaptic δ subunit-containing GABA_A_Rs [108, 109]. Importantly, these changes have been linked to altered tonic inhibition and seizure susceptibility, anxiety, and depression [108, 109].

It is accepted that women are more likely to develop anxiety and depression than men [10, 110]. Benzodiazepines, allosteric modulators of GABA_A_R function, are widely used as therapeutic agents for the treatment of anxiety [111], depression [112], and insomnia [113]. The elderly are more sensitive to the side effects of benzodiazepines, and poisoning may occur as a result of long-term use [114]. We found significant age-specific differences in the expression of GAD65 and the GABA_A_R α3 subunit, as well as sex-specific differences in GABA_A_R α1, α2, α5, and β3 subunit expression levels in the STG. This suggests that the well-established α1/2/5β2/3γ2 subunit containing benzodiazepine-sensitive receptors are upregulated in males. These findings highlight that besides differences in drug absorption, bioavailability, distribution, metabolism, and hormone balance between the sexes and between age groups [114–116], sex- and age-specific alterations in GABAergic signaling components throughout the brain should be considered in the use and prescription of benzodiazepines as they might influence the effect of these agents.

GABA_B_Rs did not show expression level differences between sexes and age groups in any of the brain regions examined. The reason why the GABA_B_Rs are spared is not known mainly due to the limited number of studies in the field. However importantly, in other brain regions, these receptors might be affected by aging or display sex-specific expression, and the robustness observed in our study might not be a general phenomenon. For example, in the macaque visual cortex, the GABA_B_R R2 subunit is upregulated with age [41].

GABA transporters are essential for the maintenance of GABA levels in the synaptic cleft. We have found that younger females have significantly lower GAT1 expression compared with younger males in the ITG. GAT1 expression in the cerebellum displayed a significant sex difference, with the older female population displaying significantly higher levels of GAT1 expression than older males. GAT1 knock-out mice exhibit prolonged inhibitory post synaptic currents in cerebellar granule cells due to reduced GABA clearance from the synaptic cleft and symptoms of ataxia, disturbed thermoregulation, and circadian rhythm and tremor [117]. In comparison to GAT1 expression, we found that GAT3 showed different expression pattern in the cerebellar cortex and sensory cortex. Older females with the highest cerebellar GAT1 expression showed the lowest GAT3 levels, and older males with the lowest cerebellar GAT1 levels showed the highest GAT3 expression. High GAT1 expression is observed on interneurons whereas GAT3 is expressed mainly on astrocytes [9, 118]. These results suggest that the GAT3 upregulation in astrocytes might occur as a compensatory mechanism, but future studies using cell-type-specific markers have to be performed to test this hypothesis. Previous studies conducted in the developing mouse [119] and in perinatal hypoxia [15, 120], schizophrenia [121], and Alzheimer’s disease [9] reported similar compensatory mechanisms, and these are essential for the maintenance of GABA levels in the synapse.

In neurodegenerative disorders like Alzheimer’s disease, sex difference has been well documented. The mechanisms underlying AD are not well understood, but aging is considered to be the leading risk factor for the disease [60–62, 110, 122–124]. Sex- and age-specific changes in key molecular components of the major transmitter systems, as described in this study, could account for the effects of sex and age on the disease, or they might be factors that influence disease prevalence and progression. In Alzheimer’s disease, the GABAergic system also undergoes significant remodeling. The STG shows downregulation of GABA_A_R α2 and α5 subunits, and the sex-specific downregulation of these receptors in females might be implicated in disease susceptibility and the faster disease progression observed within the female population [8, 9, 27, 67]. The lack of a clear understanding of sex- and age-related disease pathology in neurodegenerative diseases, as well as in other neurological disorders, like schizophrenia, epilepsy, depression, and anxiety, suggests for the importance of the inclusion of sex and age as case selection criteria or experimental parameter in the design and interpretation of all such studies, to prevent the effect of these parameters as confounding factors and to aid in improving our knowledge of the etiology, progression, and treatment of these disorders.

## Conclusions

Aging is associated with molecular, cellular, and structural changes in the brain leading to functional changes, cognitive decline, and increased vulnerability to neurological diseases, neurodegenerative conditions, sensory retrogression, and depression, just to name a few. Our study highlights that age-related GABAergic changes are brain region specific; most cortical areas are not affected. However, in the temporal lobe, we identified dramatic GABA_A_R subunit and GAD65 expression changes besides several sex-specific differences. With increasing life expectancy and the dramatically growing elderly population, understanding the mechanism and consequences of aging is critically important. There is also growing evidence that GABAergic system-specific sex differences might influence disease prevalence and progression and possibly has to be considered when designing new preventive and therapeutic options for these conditions.

## Abbreviations

AD: Alzheimer’s disease
EDTA: Ethylenediaminetetraacetic acid
GABA: Gamma-aminobutyric acid
GABA_A_R: GABA type A receptor
GABA_B_R: GABA type B receptor
GABAR: GABA receptor
GAD: Glutamic acid decarboxylase
GAT: GABA transporter
ITG: Inferior temporal gyrus
MTG: Middle temporal gyrus
OF: Older female
OM: Older male
PBS: Phosphate-buffered saline
PBST: Phosphate-buffered saline, 0.2% Triton X-100
PFC: Prefrontal cortex
SDS: Sodium dodecyl sulfate
STG: Superior temporal gyrus
TBST: Tris-buffered saline, 0.1% Tween
YF: Younger female
YM: Younger male

## References

[1] Sahara S, Yanagawa Y, O'Leary DD, Stevens CF (2012). The fraction of cortical GABAergic neurons is constant from near the start of cortical neurogenesis to adulthood. J Neurosci.

[2] Loerch PM, Lu T, Dakin KA, Vann JM, Isaacs A, Geula C, Wang J, Pan Y, Gabuzda DH, Li C, Prolla TA, Yankner BA (2008). Evolution of the aging brain transcriptome and synaptic regulation. PLoS One.

[3] McQuail JA, Frazier CJ, Bizon JL (2015). Molecular aspects of age-related cognitive decline: the role of GABA signaling. Trends Mol Med.

[4] Rozycka A, Liguz-Lecznar M (2017). The space where aging acts: focus on the GABAergic synapse. Aging Cell.

[5] Barth C, Villringer A, Sacher J (2015). Sex hormones affect neurotransmitters and shape the adult female brain during hormonal transition periods. Front Neurosci.

[6] Paganini-Hill A, Henderson VW (1994). Estrogen deficiency and risk of Alzheimer’s disease in women. Am J Epidemiol.

[7] Rissman RA, Mobley WC (2011). Implications for treatment: GABAA receptors in aging, Down syndrome and Alzheimer’s disease. J Neurochem.

[8] Kwakowsky A, Calvo-Flores Guzman B, Pandya M, Turner C, Waldvogel HJ, Faull RL (2018). GABAA receptor subunit expression changes in the human Alzheimer’s disease hippocampus, subiculum, entorhinal cortex and superior temporal gyrus. J Neurochem.

[9] Fuhrer TE, Palpagama TH, Waldvogel HJ, Synek BJL, Turner C, Faull RL, Kwakowsky A (2017). Impaired expression of GABA transporters in the human Alzheimer’s disease hippocampus, subiculum, entorhinal cortex and superior temporal gyrus. Neuroscience.

[10] Pigott TA (1999). Gender differences in the epidemiology and treatment of anxiety disorders. J Clin Psychiatry.

[11] Angermeyer MC, Kuhn L, Goldstein JM (1990). Gender and the course of schizophrenia: differences in treated outcomes. Schizophr Bull.

[12] Sharashenidze N, Schacht J, Kevanishvili Z (2007). Age-related hearing loss: gender differences. Georgian Med News.

[13] He X, Koo BB, Killiany RJ (2016). Edited magnetic resonance spectroscopy detects an age-related decline in nonhuman primate brain GABA levels. Biomed Res Int.

[14] Long Z, Medlock C, Dzemidzic M, Shin YW, Goddard AW, Dydak U (2013). Decreased GABA levels in anterior cingulate cortex/medial prefrontal cortex in panic disorder. Prog Neuro-Psychopharmacol Biol Psychiatry.

[15] Porges EC, Woods AJ, Edden RA, Puts NA, Harris AD, Chen H, Garcia AM, Seider TR, Lamb DG, Williamson JB, Cohen RA (2017). Frontal gamma-aminobutyric acid concentrations are associated with cognitive performance in older adults. Biol Psychiatry Cogn Neurosci Neuroimaging.

[16] Giorgio A, Santelli L, Tomassini V, Bosnell R, Smith S, De Stefano N, Johansen-Berg H (2010). Age-related changes in grey and white matter structure throughout adulthood. NeuroImage.

[17] Hafkemeijer A, Altmann-Schneider I, de Craen AJ, Slagboom PE, van der Grond J, Rombouts SA (2014). Associations between age and gray matter volume in anatomical brain networks in middle-aged to older adults. Aging Cell.

[18] Liguz-Lecznar M, Lehner M, Kaliszewska A, Zakrzewska R, Sobolewska A, Kossut M (2015). Altered glutamate/GABA equilibrium in aged mice cortex influences cortical plasticity. Brain Struct Funct.

[19] Banuelos C, Beas BS, McQuail JA, Gilbert RJ, Frazier CJ, Setlow B, Bizon JL (2014). Prefrontal cortical GABAergic dysfunction contributes to age-related working memory impairment. J Neurosci.

[20] Sigel E, Steinmann ME (2012). Structure, function, and modulation of GABA(A) receptors. J Biol Chem.

[21] Chen ZW, Olsen RW (2007). GABAA receptor associated proteins: a key factor regulating GABAA receptor function. J Neurochem.

[22] Sieghart W, Fuchs K, Tretter V, Ebert V, Jechlinger M, Hoger H, Adamiker D (1999). Structure and subunit composition of GABA(A) receptors. Neurochem Int.

[23] Sieghart W, Savic MM (2018). International Union of Basic and Clinical Pharmacology. CVI: GABAA receptor subtype- and function-selective ligands: key issues in translation to humans. Pharmacol Rev.

[24] McKernan RM, Whiting PJ (1996). Which GABAA-receptor subtypes really occur in the brain?. Trends Neurosci.

[25] Olsen RW, Sieghart W (2009). GABA A receptors: subtypes provide diversity of function and pharmacology. Neuropharmacology.

[26] Caspary DM, Hughes LF, Ling LL (2013). Age-related GABAA receptor changes in rat auditory cortex. Neurobiol Aging.

[27] Govindpani K, Calvo-Flores Guzman B, Vinnakota C, Waldvogel HJ, Faull RL, Kwakowsky A. Towards a better understanding of GABAergic remodeling in Alzheimer’s disease. Int J Mol Sci. 2017;18(8):1813 10.3390/ijms18081813 PMC5578199 28825683

[28] Hill DR, Bowery NG (1981). 3H-baclofen and 3H-GABA bind to bicuculline-insensitive GABA B sites in rat brain. Nature.

[29] Jones KA, Borowsky B, Tamm JA, Craig DA, Durkin MM, Dai M, Yao WJ, Johnson M, Gunwaldsen C, Huang LY, Tang C, Shen Q, Salon JA, Morse K, Laz T, Smith KE, Nagarathnam D, Noble SA, Branchek TA, Gerald C (1998). GABA(B) receptors function as a heteromeric assembly of the subunits GABA(B)R1 and GABA(B)R2. Nature.

[30] Kaupmann K, Malitschek B, Schuler V, Heid J, Froestl W, Beck P, Mosbacher J, Bischoff S, Kulik A, Shigemoto R, Karschin A, Bettler B (1998). GABA(B)-receptor subtypes assemble into functional heteromeric complexes. Nature.

[31] Beas BS, McQuail JA, Ban Uelos C, Setlow B, Bizon JL (2017). Prefrontal cortical GABAergic signaling and impaired behavioral flexibility in aged F344 rats. Neuroscience.

[32] Jurado-Parras MT, Delgado-Garcia JM, Sanchez-Campusano R, Gassmann M, Bettler B, Gruart A (2016). Presynaptic GABAB receptors regulate hippocampal synapses during associative learning in behaving mice. PLoS One.

[33] McQuail JA, Banuelos C, LaSarge CL, Nicolle MM, Bizon JL (2012). GABA(B) receptor GTP-binding is decreased in the prefrontal cortex but not the hippocampus of aged rats. Neurobiol Aging.

[34] Lasarge CL, Banuelos C, Mayse JD, Bizon JL (2009). Blockade of GABA(B) receptors completely reverses age-related learning impairment. Neuroscience.

[35] Milbrandt JC, Albin RL, Caspary DM (1994). Age-related decrease in GABAB receptor binding in the Fischer 344 rat inferior colliculus. Neurobiol Aging.

[36] Turgeon SM, Albin RL (1994). GABAB binding sites in early adult and aging rat brain. Neurobiol Aging.

[37] Caspary DM, Milbrandt JC, Helfert RH (1995). Central auditory aging: GABA changes in the inferior colliculus. Exp Gerontol.

[38] Jin XT, Galvan A, Wichmann T, Smith Y (2011). Localization and function of GABA transporters GAT-1 and GAT-3 in the basal ganglia. Front Syst Neurosci.

[39] Scimemi A (2014). Structure, function, and plasticity of GABA transporters. Front Cell Neurosci.

[40] Sundman-Eriksson I, Allard P (2006). Age-correlated decline in [3H] tiagabine binding to GAT-1 in human frontal cortex. Aging Clin Exp Res.

[41] Liao C, Han Q, Ma Y, Su B (2016). Age-related gene expression change of GABAergic system in visual cortex of rhesus macaque. Gene.

[42] Schmolesky MT, Wang Y, Pu M, Leventhal AG (2000). Degradation of stimulus selectivity of visual cortical cells in senescent rhesus monkeys. Nat Neurosci.

[43] O'Gorman RL, Michels L, Edden RA, Murdoch JB, Martin E (2011). In vivo detection of GABA and glutamate with MEGA-PRESS: reproducibility and gender effects. J Magn Reson Imaging.

[44] Backstrom T, Haage D, Lofgren M, Johansson IM, Stromberg J, Nyberg S, Andreen L, Ossewaarde L, van Wingen GA, Turkmen S, Bengtsson SK (2011). Paradoxical effects of GABA-A modulators may explain sex steroid induced negative mood symptoms in some persons. Neuroscience.

[45] Epperson CN, O'Malley S, Czarkowski KA, Gueorguieva R, Jatlow P, Sanacora G, Rothman DL, Krystal JH, Mason GF (2005). Sex, GABA, and nicotine: the impact of smoking on cortical GABA levels across the menstrual cycle as measured with proton magnetic resonance spectroscopy. Biol Psychiatry.

[46] De Bondt T, De Belder F, Vanhevel F, Jacquemyn Y, Parizel PM (2015). Prefrontal GABA concentration changes in women-influence of menstrual cycle phase, hormonal contraceptive use, and correlation with premenstrual symptoms. Brain Res.

[47] Kwakowsky A, Herbison AE, Abraham IM (2012). The role of cAMP response element-binding protein in estrogen negative feedback control of gonadotropin-releasing hormone neurons. J Neurosci.

[48] Malyala A, Kelly MJ, Ronnekleiv OK (2005). Estrogen modulation of hypothalamic neurons: activation of multiple signaling pathways and gene expression changes. Steroids.

[49] Kwakowsky A, Cheong RY, Herbison AE, Abraham IM (2014). Non-classical effects of estradiol on cAMP responsive element binding protein phosphorylation in gonadotropin-releasing hormone neurons: mechanisms and role. Front Neuroendocrinol.

[50] Nilsson S, Makela S, Treuter E, Tujague M, Thomsen J, Andersson G, Enmark E, Pettersson K, Warner M, Gustafsson JA (2001). Mechanisms of estrogen action. Physiol Rev.

[51] Abraham IM, Herbison AE (2005). Major sex differences in non-genomic estrogen actions on intracellular signaling in mouse brain in vivo. Neuroscience.

[52] Vasudevan N, Pfaff DW (2008). Non-genomic actions of estrogens and their interaction with genomic actions in the brain. Front Neuroendocrinol.

[53] Micevych P, Dominguez R (2009). Membrane estradiol signaling in the brain. Front Neuroendocrinol.

[54] Romano N, Lee K, Abraham IM, Jasoni CL, Herbison AE (2008). Nonclassical estrogen modulation of presynaptic GABA terminals modulates calcium dynamics in gonadotropin-releasing hormone neurons. Endocrinology.

[55] Herbison AE, Heavens RP, Dyer RG (1990). Oestrogen modulation of excitatory A1 noradrenergic input to rat medial preoptic gamma aminobutyric acid neurones demonstrated by microdialysis. Neuroendocrinology.

[56] Calvo-Flores Guzman B, Vinnakota C, Govindpani K, Waldvogel H, Faull RL, Kwakowsky A (2018). The GABAergic system as a therapeutic target for Alzheimer’s disease. J Neurochem.

[57] Smith SS (2002). Withdrawal properties of a neuroactive steroid: implications for GABA(A) receptor gene regulation in the brain and anxiety behavior. Steroids.

[58] Gulinello M, Gong QH, Li X, Smith SS (2001). Short-term exposure to a neuroactive steroid increases alpha4 GABA(A) receptor subunit levels in association with increased anxiety in the female rat. Brain Res.

[59] Hantsoo L, Epperson CN (2015). Premenstrual dysphoric disorder: epidemiology and treatment. Curr Psychiatry Rep.

[60] Kwakowsky A, Milne MR, Waldvogel HJ, Faull RL (2016). Effect of estradiol on Neurotrophin receptors in basal forebrain cholinergic neurons: relevance for Alzheimer’s disease. Int J Mol Sci.

[61] Milne MR, Haug CA, Abraham IM, Kwakowsky A (2015). Estradiol modulation of neurotrophin receptor expression in female mouse basal forebrain cholinergic neurons in vivo. Endocrinology.

[62] Kwakowsky A, Koszegi Z, Cheong RY, Abraham IM (2013). Neuroprotective effects of non-classical estrogen-like signaling activators: from mechanism to potential implications. CNS Neurol Disord Drug Targets.

[63] Kwakowsky A, Potapov K, Kim S, Peppercorn K, Tate WP, Abraham IM (2016). Treatment of beta amyloid 1-42 (Abeta (1-42))-induced basal forebrain cholinergic damage by a non-classical estrogen signaling activator in vivo. Sci Rep.

[64] Flores-Ramos M, Salinas M, Carvajal-Lohr A, Rodriguez-Bores L (2017). The role of gamma-aminobutyric acid in female depression. Gac Med Mex.

[65] Fee C, Banasr M, Sibille E (2017). Somatostatin-positive gamma-aminobutyric acid interneuron deficits in depression: cortical microcircuit and therapeutic perspectives. Biol Psychiatry.

[66] Waldvogel HJ, Curtis MA, Baer K, Rees MI, Faull RL (2006). Immunohistochemical staining of post-mortem adult human brain sections. Nat Protoc.

[67] Kwakowsky A, Calvo-Flores Guzmán B, Govindpani K, Waldvogel HJ, Faull RLM (2018). GABAA receptors in Alzheimer’s disease: highly localized remodeling of a complex and diverse signaling pathway. Neural Regener Res.

[68] Fritschy JM (2008). Epilepsy, E/I balance and GABA(A) receptor plasticity. Front Mol Neurosci.

[69] Kralic JE, Sidler C, Parpan F, Homanics GE, Morrow AL, Fritschy JM (2006). Compensatory alteration of inhibitory synaptic circuits in cerebellum and thalamus of gamma-aminobutyric acid type A receptor alpha1 subunit knockout mice. J Comp Neurol.

[70] Eran A, Hodes A, Izbudak I (2016). Bilateral temporal lobe disease: looking beyond herpes encephalitis. Insights Imaging.

[71] Kiernan JA (2012). Anatomy of the temporal lobe. Epilepsy Res Treat.

[72] Cipolloni PB, Pandya DN (1985). Topography and trajectories of commissural fibers of the superior temporal region in the rhesus monkey. Exp Brain Res.

[73] Gultekin SH, Rosenfeld MR, Voltz R, Eichen J, Posner JB, Dalmau J (2000). Paraneoplastic limbic encephalitis: neurological symptoms, immunological findings and tumour association in 50 patients. Brain.

[74] Arendt T, Bruckner MK, Gertz HJ, Marcova L (1998). Cortical distribution of neurofibrillary tangles in Alzheimer’s disease matches the pattern of neurons that retain their capacity of plastic remodelling in the adult brain. Neuroscience.

[75] Pinto JG, Hornby KR, Jones DG, Murphy KM (2010). Developmental changes in GABAergic mechanisms in human visual cortex across the lifespan. Front Cell Neurosci.

[76] Asada H, Kawamura Y, Maruyama K, Kume H, Ding R, Ji FY, Kanbara N, Kuzume H, Sanbo M, Yagi T, Obata K (1996). Mice lacking the 65 kDa isoform of glutamic acid decarboxylase (GAD65) maintain normal levels of GAD67 and GABA in their brains but are susceptible to seizures. Biochem Biophys Res Commun.

[77] Kash SF, Johnson RS, Tecott LH, Noebels JL, Mayfield RD, Hanahan D, Baekkeskov S (1997). Epilepsy in mice deficient in the 65-kDa isoform of glutamic acid decarboxylase. Proc Natl Acad Sci U S A.

[78] Heldt SA, Green A, Ressler KJ (2004). Prepulse inhibition deficits in GAD65 knockout mice and the effect of antipsychotic treatment. Neuropsychopharmacology.

[79] Muller I, Caliskan G, Stork O (2015). The GAD65 knock out mouse - a model for GABAergic processes in fear- and stress-induced psychopathology. Genes Brain Behav.

[80] Qi J, Kim M, Sanchez R, Ziaee SM, Kohtz JD, Koh S (2018). Enhanced susceptibility to stress and seizures in GAD65 deficient mice. PLoS One.

[81] Wu H, Jin Y, Buddhala C, Osterhaus G, Cohen E, Jin H, Wei J, Davis K, Obata K, Wu JY (2007). Role of glutamate decarboxylase (GAD) isoform, GAD65, in GABA synthesis and transport into synaptic vesicles-Evidence from GAD65-knockout mice studies. Brain Res.

[82] Henneberger C, Kirischuk S, Grantyn R (2005). Brain-derived neurotrophic factor modulates GABAergic synaptic transmission by enhancing presynaptic glutamic acid decarboxylase 65 levels, promoting asynchronous release and reducing the number of activated postsynaptic receptors. Neuroscience.

[83] Lau CG, Murthy VN (2012). Activity-dependent regulation of inhibition via GAD67. J Neurosci.

[84] Fillman SG, Duncan CE, Webster MJ, Elashoff M, Weickert CS (2010). Developmental co-regulation of the beta and gamma GABAA receptor subunits with distinct alpha subunits in the human dorsolateral prefrontal cortex. Int J Dev Neurosci.

[85] Hughes LF, Turner JG, Parrish JL, Caspary DM (2010). Processing of broadband stimuli across A1 layers in young and aged rats. Hear Res.

[86] Ling LL, Hughes LF, Caspary DM (2005). Age-related loss of the GABA synthetic enzyme glutamic acid decarboxylase in rat primary auditory cortex. Neuroscience.

[87] Silbersweig DA, Stern E, Frith C, Cahill C, Holmes A, Grootoonk S, Seaward J, McKenna P, Chua SE, Schnorr L (1995). A functional neuroanatomy of hallucinations in schizophrenia. Nature.

[88] Kim JJ, Crespo-Facorro B, Andreasen NC, O'Leary DS, Magnotta V, Nopoulos P (2003). Morphology of the lateral superior temporal gyrus in neuroleptic nai;ve patients with schizophrenia: relationship to symptoms. Schizophr Res.

[89] Gaser C, Nenadic I, Volz HP, Buchel C, Sauer H (2004). Neuroanatomy of “hearing voices”: a frontotemporal brain structural abnormality associated with auditory hallucinations in schizophrenia. Cereb Cortex.

[90] van Tol MJ, van der Meer L, Bruggeman R, Modinos G, Knegtering H, Aleman A (2014). Voxel-based gray and white matter morphometry correlates of hallucinations in schizophrenia: the superior temporal gyrus does not stand alone. Neuroimage Clin.

[91] Deng C, Huang XF (2006). Increased density of GABAA receptors in the superior temporal gyrus in schizophrenia. Exp Brain Res.

[92] Pierri JN, Chaudry AS, Woo TU, Lewis DA (1999). Alterations in chandelier neuron axon terminals in the prefrontal cortex of schizophrenic subjects. Am J Psychiatry.

[93] Volk DW, Pierri JN, Fritschy JM, Auh S, Sampson AR, Lewis DA (2002). Reciprocal alterations in pre- and postsynaptic inhibitory markers at chandelier cell inputs to pyramidal neurons in schizophrenia. Cereb Cortex.

[94] Hoftman GD, Volk DW, Bazmi HH, Li S, Sampson AR, Lewis DA (2015). Altered cortical expression of GABA-related genes in schizophrenia: illness progression vs developmental disturbance. Schizophr Bull.

[95] Hashimoto T, Arion D, Unger T, Maldonado-Aviles JG, Morris HM, Volk DW, Mirnics K, Lewis DA (2008). Alterations in GABA-related transcriptome in the dorsolateral prefrontal cortex of subjects with schizophrenia. Mol Psychiatry.

[96] Duncan CE, Webster MJ, Rothmond DA, Bahn S, Elashoff M, Shannon Weickert C (2010). Prefrontal GABA(A) receptor alpha-subunit expression in normal postnatal human development and schizophrenia. J Psychiatr Res.

[97] Lindamer LA, Bailey A, Hawthorne W, Folsom DP, Gilmer TP, Garcia P, Hough RL, Jeste DV (2003). Gender differences in characteristics and service use of public mental health patients with schizophrenia. Psychiatr Serv.

[98] Leung A, Chue P (2000). Sex differences in schizophrenia, a review of the literature. Acta Psychiatr Scand Suppl.

[99] Salokangas RK (1983). Prognostic implications of the sex of schizophrenic patients. Br J Psychiatry.

[100] McGrath J, Saha S, Chant D, Welham J (2008). Schizophrenia: a concise overview of incidence, prevalence, and mortality. Epidemiol Rev.

[101] Thomas P, Wood J, Chandra A, Nimgaonkar VL, Deshpande SN (2010). Differences among men and women with schizophrenia: a study of US and Indian samples. Psychiatry Investig.

[102] Hafner H, Riecher-Rossler A, An Der Heiden W, Maurer K, Fatkenheuer B, Loffler W (1993). Generating and testing a causal explanation of the gender difference in age at first onset of schizophrenia. Psychol Med.

[103] Mohler H (2006). GABAA receptors in central nervous system disease: anxiety, epilepsy, and insomnia. J Recept Signal Transduct Res.

[104] Christensen J, Kjeldsen MJ, Andersen H, Friis ML, Sidenius P (2005). Gender differences in epilepsy. Epilepsia.

[105] Janszky J, Schulz R, Janszky I, Ebner A (2004). Medial temporal lobe epilepsy: gender differences. J Neurol Neurosurg Psychiatry.

[106] Herzog AG, Klein P, Ransil BJ (1997). Three patterns of catamenial epilepsy. Epilepsia.

[107] Sperk G, Furtinger S, Schwarzer C, Pirker S (2004). GABA and its receptors in epilepsy. Adv Exp Med Biol.

[108] Maguire JL, Stell BM, Rafizadeh M, Mody I (2005). Ovarian cycle-linked changes in GABA(A) receptors mediating tonic inhibition alter seizure susceptibility and anxiety. Nat Neurosci.

[109] Maguire J, Mody I (2008). GABA(A)R plasticity during pregnancy: relevance to postpartum depression. Neuron.

[110] Breslau N, Schultz L, Peterson E (1995). Sex differences in depression: a role for preexisting anxiety. Psychiatry Res.

[111] Vajda FJ, Burrows GD (1983). Use of drugs in the treatment of anxiety. Aust Fam Physician.

[112] Johnson DA (1985). The use of benzodiazepines in depression. Br J Clin Pharmacol.

[113] Simon GE, VonKorff M (1997). Prevalence, burden, and treatment of insomnia in primary care. Am J Psychiatry.

[114] Klein-Schwartz W, Oderda GM (1991). Poisoning in the elderly. Epidemiological, clinical and management considerations. Drugs Aging.

[115] Wilson K (1984). Sex-related differences in drug disposition in man. Clin Pharmacokinet.

[116] Nikaido AM, Ellinwood EH, Heatherly DG, Gupta SK (1990). Age-related increase in CNS sensitivity to benzodiazepines as assessed by task difficulty. Psychopharmacology.

[117] Chiu CS, Brickley S, Jensen K, Southwell A, McKinney S, Cull-Candy S, Mody I, Lester HA (2005). GABA transporter deficiency causes tremor, ataxia, nervousness, and increased GABA-induced tonic conductance in cerebellum. J Neurosci.

[118] Zhou Y, Danbolt NC (2013). GABA and glutamate transporters in Brain. Front Endocrinol (Lausanne).

[119] Kwakowsky A, Schwirtlich M, Kooy F, Abraham I, Mate Z, Katarova Z, Szabo G (2008). GABA neurotransmitter signaling in the developing mouse lens: dynamic regulation of components and functionality. Dev Dyn.

[120] Pozdnyakova N, Dudarenko M, Yatsenko L, Himmelreich N, Krupko O, Borisova T (2014). Perinatal hypoxia: different effects of the inhibitors of GABA transporters GAT1 and GAT3 on the initial velocity of [3H]GABA uptake by cortical, hippocampal, and thalamic nerve terminals. Croat Med J.

[121] Schleimer SB, Hinton T, Dixon G, Johnston GA (2004). GABA transporters GAT-1 and GAT-3 in the human dorsolateral prefrontal cortex in schizophrenia. Neuropsychobiology.

[122] Ferretti MT, Iulita MF, Cavedo E, Chiesa PA, Schumacher Dimech A, Santuccione Chadha A, Baracchi F, Girouard H, Misoch S, Giacobini E, Depypere H, Hampel H, Women’s Brain Project and, the Alzheimer Precision Medicine I (2018). Sex differences in Alzheimer disease - the gateway to precision medicine. Nat Rev Neurol.

[123] Nebel RA, Aggarwal NT, Barnes LL, Gallagher A, Goldstein JM, Kantarci K, Mallampalli MP, Mormino EC, Scott L, Yu WH, Maki PM, Mielke MM (2018). Understanding the impact of sex and gender in Alzheimer’s disease: a call to action. Alzheimers Dement.

[124] Altemus M, Sarvaiya N, Neill Epperson C (2014). Sex differences in anxiety and depression clinical perspectives. Front Neuroendocrinol.

